# Influence of infection on the distribution patterns of NIH-Chronic Prostatitis Symptom Index scores in patients with chronic prostatitis/chronic pelvic pain syndrome (CP/CPPS)

**DOI:** 10.3892/etm.2013.1174

**Published:** 2013-06-21

**Authors:** V. MAGRI, F.M.E. WAGENLEHNER, E. MARRAS, J.W.O. VAN TILL, J. HOUBIERS, P. PANAGOPOULOS, G.L. PETRIKKOS, G. PERLETTI

**Affiliations:** 1 Urology and Sonography Outpatient Clinic, Azienda Ospedaliera Istituti Clinici di Perfezionamento, Milan, Italy;; 2 Department of Urology, Pediatric Urology and Andrology, Justus-Liebig-University, Giessen, Germany;; 3 Biomedical Research Division, Department of Theoretical and Applied Sciences, Università degli Studi dell’Insubria, Busto Arsizio/Varese, Italy;; 4 Global Medical Science, Astellas Pharma Europe, Leiderdorp, The Netherlands;; 5 Second Department of Internal Medicine, General University Hospital of Alexandroupolis, Democritus University of Thrace, Alexandroupolis;; 6 Fourth Department of Internal Medicine, Attikon University General Hospital, National Kapodistrian University of Athens, Athens, Greece;; 7 Department of Basic Medical Sciences, Faculty of Medicine and Health Sciences, Ghent University, Ghent, Belgium

**Keywords:** chronic prostatitis/chronic pelvic pain syndrome, chronic prostatitis, chronic bacterial prostatitis, UPOINT, phenotyping, prostate infection, LUTS, chronic prostatitis symptom index

## Abstract

Chronic prostatitis/chronic pelvic pain syndrome (CP/CPPS) is a complex condition for which the etiological determinants are still poorly defined. To better characterize the diagnostic and therapeutic profile of patients, an algorithm known as UPOINT was created, addressing six major phenotypic domains of CP/CPPS, specifically the urinary (U), psycho-social (P), organ-specific (O), infection (I), neurological/systemic (N) and muscular tenderness (T) domains. An additional sexual dysfunction domain may be included in the UPOINT(S) system. The impact of the infection domain on the severity of CP/CPPS symptoms is a controversial issue, due to the contradictory results of different trials. The aim of the present retrospective study was to further analyze the extent to which a positive infection domain of UPOINTS may modify the pattern of CP/CPPS symptom scores, assessed with the National Institutes of Health-Chronic Prostatitis Symptom Index (NIH-CPSI). In a cohort of 935 patients that was divided on the basis of the presence or absence of prostatic infection, more severe clinical symptoms were shown by the patients with infection (median NIH total score: 24 versus 20 points in uninfected patients; P<0.001). Moreover, NIH-CPSI score distribution curves were shifted towards more severe symptoms in patients with a positive infection domain. Division of the patients into the six most prominent phenotypic clusters of UPOINTS revealed that the ‘prostate infection-related sexual dysfunction’ cluster, including the highest proportion of patients with evidence of infection (80%), scored the highest number of NIH-CPSI points among all the clusters. To assess the influence of the infection domain on the severity of patients’ symptoms, all subjects with evidence of infection were withdrawn from the ‘prostate infection-related sexual dysfunction’ cluster. This modified cluster showed symptom scores significantly less severe than the original cluster, and the CPSI values became comparable to the scores of the five other clusters, which were virtually devoid of patients with evidence of infection. These results suggest that the presence of pathogens in the prostate gland may significantly affect the clinical presentation of patients affected by CP/CPPS, and that the infection domain may be a determinant of the severity of CP/CPPS symptoms in clusters of patients phenotyped with the UPOINTS system. This evidence may convey considerable therapeutic implications.

## Introduction

Chronic prostatitis/chronic pelvic pain syndrome (CP/CPPS) is a complex condition for which the etiological determinants are still poorly defined. Its prevalence ranges between 2.2 and 13.8%, depending on specific populations, countries and surveys (1,2). CP/CPPS is characterized by a wide array of symptoms, including pain in the pelvic region, irritative and/or obstructive voiding symptoms, other pelvic disturbances, sexual dysfunction and psycho-social maladjustment amongst others (3).

Due to the fact that every patient with CP/CPPS presents a unique, individual profile of symptoms defining the syndrome, phenotypic characterization is required to profile the clinical features of each single subject, and to direct symptomatic therapy. For this reason, a novel diagnostic/therapeutic algorithm known as UPOINT was created, addressing six major phenotypic domains of CP/CPPS, specifically the urinary (U), psycho-social (P), organ-specific (O), infection (I), neurological/systemic (N) and muscular tenderness (T) domains. UPOINT, with or without an additional domain addressing sexual dysfunction (‘UPOINTS’), has been initially validated in two cohort studies, performed on North-American (4) and European patients (5).

The major finding of the first North-American validation study was the correlation between the number of UPOINT domains and the total score of the National Institutes of Health-Chronic Prostatitis Symptom Index (NIH-CPSI) measured in each patient (4). Whereas a strong correlation was also observed in the European cohort (5), certain discrepancies between the two studies were evident. In the study by Shoskes *et al*, the severity of the symptoms in the patients with a positive infection domain was not significantly different from the symptom scores of patients without evidence of prostatic infection (4). Conversely, in the study by Magri *et al*, patients with a positive infection domain showed higher symptom scores, measured using the NIH-CPSI (5). This suggested the existence of different populations of patients, exhibiting different degrees of symptom severity, depending on the presence or absence of infection. Recently, we have investigated the distribution of NIH-CPSI symptom scores in a large cohort of patients from different countries globally (6). Graphic representations of the total NIH-CPSI symptom score distributions showed irregular patterns (6), possibly due to multiple overlapping peaks, which suggested the existence of different subpopulations of patients showing various degrees of symptom severity.

In the present study, we report the results of a retrospective analysis of a population of 935 Italian patients who had been included in the large international studies mentioned previously (5,6). The aim of the present study was to investigate in greater detail the extent to which a positive UPOINTS infection domain may modify the distribution patterns of NIH-CPSI symptom scores in this patient population.

## Patients and methods

### Clinical database

The present retrospective observational study was performed on a database of 935 Italian patients, representing a subset of a total population of 1,563 subjects from Europe and North America enrolled in two recent, ethically approved international studies investigating NIH-CPSI and the UPOINT(S) system (5,6). This subset of patients was diagnosed and treated by one urologist (VM) in a single specialized, second referral prostatitis center in Italy (Urology and Urological Sonography Secondary Care Clinic, Azienda Ospedaliera Istituti Clinici di Perfezionamento, Milan). Patients provided written consent for the handling and anonymous publication of their data.

The database used for this subset analysis included demographic data, diagnosis [CP/CPPS, category IIIa (inflammatory) versus IIIb (non-inflammatory)], UPOINT phenotypes and the scores of the NIH-CPSI questionnaire.

### Inclusion criteria and diagnostic procedures

CP/CPPS was diagnosed according to the NIH criteria (7). Inclusion/exclusion criteria for the study, as well as the items by which patients were allocated as positive for the individual UPOINT domains, have been described in the main study report (5). Exclusion criteria included a diagnosis of acute or chronic bacterial prostatitis (CBP; NIH categories I or II, NIH criteria) (7).

The presence of bacteria or pathogens in the prostate was assessed with the ‘4-glass’ lower urinary tract segmented test according to Meares and Stamey, based on the collection and culture of i) first-voided (VB1), ii) midstream (VB2) and iii) post-massage voided urine (VB3) specimens, and iv) of expressed prostatic secretions (EPS) collected during prostatic massage (8). A one-log ratio between bacterial counts in VB3 versus VB2 or VB1 was the cutoff value defining “prostatic infection (8) and a positive UPOINTS infection domain.

CP/CPPS symptoms were evaluated by asking the patients to complete the NIH-CPSI questionnaire, an internationally validated clinical tool measuring disease symptoms and characteristics (9). The NIH-CPSI measures combined aspects of the three most important symptom domains of CP/CPPS, with higher scores indicating higher disease severity. These symptom domains include pain (location, frequency and severity; score range, 0 to 21), voiding problems (irritative and obstructive symptoms; score range, 0 to 10), and impact of the disease on the quality of life of patients (QoL; score range, 0 to 12), with a total score ranging from 0 to 43.

### Statistics

χ^2^ analysis was used to evaluate differences in the proportions of patients with and without a positive infection domain for each score of the NIH-CPSI questionnaire (total score, and pain, void and QoL sub-scores).

The median was used as a measure of central tendency for the scores and sub-scores of the NIH-CPSI. The upper and lower quartiles were calculated to evaluate the dispersion of the data. The Mann-Whitney-Wilcoxon test was used to analyze unpaired differences in NIH-CPSI scores between patient groups.

Due to its large sample size, the present study was adequately powered (1−β=90%) to detect clinically relevant changes (6 points) in the total scores of the NIH-CPSI, with a 5% α error probability. The results of all statistical tests were considered significant in the presence of an α error <0.05. Data were analyzed with the XLStatistics 5.71 program (^©^Rodney Carr; http://www.deakin.edu.au/~rodneyc/).

## Results

### NIH-CPSI scores and distribution patterns in patients with or without evidence of infection

**Figure 1.**
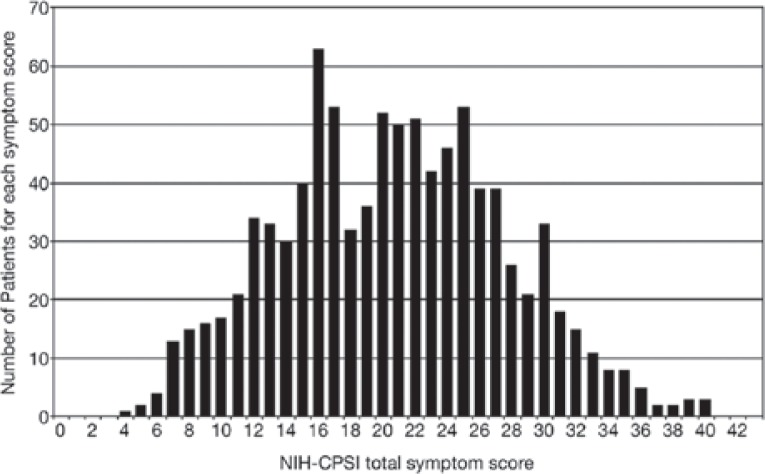
Distribution pattern of total National Institutes of Health-Chronic Prostatitis Symptom Index (NIH-CPSI) scores in the chronic prostatitis/chronic pelvic pain syndrome (CP/CPPS) patient population analyzed in this study. The vertical axis shows the total number of patients with a specific NIH-CPSI score.

Fig. 1 shows the distribution pattern of total NIH-CPSI scores in the total CP/CPPS patient population. The distribution appears to be non-unimodal and slightly right-skewed (moment coefficient of skewness, 0.11: ‘approximately symmetric’ by definition). Table I shows the median values of the NIH-CPSI total score and symptom domain subscores, and the statistical analysis of the differences between cohorts of patients with or without evidence of prostatic infection (i.e. a positive or negative UPOINTS infection domain, respectively). The pain and QoL subdomain scores, and the total score of the NIH-CPSI were significantly different between these cohorts. The voiding symptom scores of the NIH-CPSI did not differ between cohorts.

**Figure 2.**
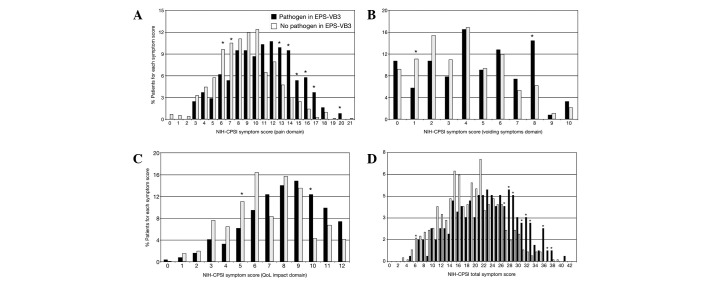
Distribution patterns of National Institutes of Health-Chronic Prostatitis Symptom Index (NIH-CPSI) scores in patients with a positive (black bars) or negative (empty bars) infection domain of the UPOINTS system [i.e. patients with positive or negative microbiological cultures of expressed prostatic secretions (EPS) and/or post-massage voided urine (VB3) specimens from a standard ‘4-glass’ test according to Meares and Stamey (8)]. (A) Pain domain; (B) voiding symptom domain; (C) impact on quality of life (QoL) domain; (D) total NIH-CPSI score. The vertical axes show the percentages of patients with a specific symptom score per-group (infection group vs. no infection group). The comparison between proportions of patients showing the same score in the two groups was performed using the χ^2^ test. ^*^P<0.05.

Fig. 2 shows the distribution patterns of NIH-CPSI scores in patients with a positive or negative infection domain of the UPOINTS phenotyping system. In the graph showing the pain domain (Fig. 2A), the distribution pattern of patients with a positive infection domain is shifted to the right, i.e. toward more severe pain symptom scores. Significantly higher proportions of patients with infection showed symptom scores as high as 13–17, compared with patients showing no evidence of bacterial infection in the prostate.

Since the pain domain is the component of greater weight in the NIH-CPSI scoring system, the previously described difference between ‘infected’ versus ‘non-infected’ patients is reflected in the distribution of the NIH-CPSI total scores, as shown in Fig. 2D. At symptom scores >27, patients with a positive infection domain were predominantly present in significantly higher proportions, compared with subjects without evidence of infection. These differences were less evident in the two other sections of the NIH-CPSI test, i.e. the voiding and QoL domains. However, significantly higher numbers of infected patients were still observed at higher scores in these two domains (Fig. 2B and C).

### Impact of infection on NIH-CPSI scores in phenotypic clusters of CP/CPPS patient

Since the distribution of data shown in Fig. 2 indicated the existence of two distinct populations with differing symptom scores, and due to the fact that infection was the discriminant element in this respect, we investigated whether it was also possible to observe these differences in the CP/CPPS phenotype clusters reported by Davis *et al* in their recent UPOINT(S) cluster analysis (10). In this previous study, it was shown that patients with CP/CPPS tended to segregate into six different clusters, each containing patients showing high frequencies of specific UPOINTS domains (10). These clusters were denominated: i) prostate infection-related sexual dysfunction (high frequencies of O, I and S domains); ii) widespread symptoms (high frequencies of U, O and N domains); iii) psychosocially complex lower urinary tract symptoms (LUTS) with sexual dysfunction (high frequencies of P and S domains); iv) psychosocially complex LUTS without sexual dysfunction (high frequencies of U and P domains); v) muscle tenderness/fibromyalgia-like symptoms (high frequency of T domain) and vi) uncomplicated LUTS (increased frequencies of U and O domains). The infection domain of the UPOINT system was present at very high frequency (80% of patients) only in the first cluster.

NIH-CPSI scores were calculated subsequent to the classification of the present patient population according to the clusters described by Davis *et al* (10). As shown in Table II, patients belonging to the ‘prostate infection-related sexual dysfunction’ cluster showed significantly higher pain, voiding symptom, QoL and total scores of the NIH-CPSI, compared with all other clusters. Table II also shows that the scores of a modified group, i.e. the ‘prostate infection-related sexual dysfunction’ cluster lacking all patients with evidence of infection, were significantly lower than the ones shown by the original cluster, and were virtually equivalent to those shown by the other clusters, which were also lacking high proportions of infected patients.

## Discussion

Category III CP/CPPS, and its inflammatory and non-inflammatory (categories IIIa and IIIb, respectively) variants, have been originally defined, diagnosed and investigated as a chronic prostatitis syndrome involving pelvic pain and voiding symptoms in the presence of sterile microbiological cultures of expressed prostatic secretions and/or urine specimens following prostatic massage (11). Over time, this original definition has evolved, and experts have proposed a model whereby the prostate of patients with CP/CPPS may be colonized by a variety of organisms, including either traditional uropathogens (such as *E.coli*, *Klebsiella spp*., other *Enterobacteriaceae* and *Enterococci*), *viz* the etiological agents of acute and chronic bacterial prostatitis, or bacteria causing disease in other distant or adjacent organs (including coagulase-negative *Staphylococci*, *Streptococci*, *Chlamydia trachomatis* and *Mycoplasmata*) that have not yet been unanimously classified as prostatic pathogens (12). This model, also based on the results of a study showing similar infection frequencies in symptomatic versus asymptomatic patients, has led to a tentative definition of ‘bacterial CP/CPPS’ (13: see reply to published letter).

According to this theory, these bacteria do not act as determinants of pathogenicity in category III CP/CPPS. Instead, they represent ‘bystander flora’, whose pharmacological eradication would not be likely to result in symptom remission. Using this model, a diagnosis of category II CBP is only possible in cases of a documented history of relapsing episodes of urinary tract infections (UTIs) originating from an infected prostate. This concept has led to the inclusion of an infection domain into the UPOINT algorithm, a system recently proposed to improve the phenotypic profiling of patients with CP/CPPS (4).

However, this concept has not been unanimously applied in clinical practice, and other clinicians and scientists have maintained that the presence of pathogens in the prostatic secretions of chronically symptomatic patients may be etiologically linked to the clinical manifestation of category II CBP, even in the absence of a long, documented history of repeated flare-ups of the infection (14,15). According to this view, a symptomatic episode of clinical chronic prostatitis with evidence of uropathogens in the prostate may represent the initial phase of a history of CBP, and there is therefore a requirement for it to be diagnosed and treated accordingly. Antibacterial treatment of such patients has been associated with symptom remission in numerous cases (14,15). For clarity, it is necessary to state that in the present study CP/CPPS was diagnosed according to the ‘bystander flora’ hypothesis, and patients not showing a documented history of repeated episodes of UTIs stemming from a chronically infected prostate were classified as being affected by category III CP/CPPS.

In the first validation study by the authors who proposed the new UPOINT system, there was a failure to demonstrate the influence of pathogens cultured in prostate-specific specimens on CP/CPPS clinical symptoms, as assessed using the NIH-CPSI questionnaire (4). By contrast, data obtained by our research group suggested that the presence of microorganisms may influence the severity of clinical symptoms of CP/CPPS. In patients showing evidence of infection at the prostatic level, the mean total NIH-CPSI symptom scores were significantly increased by ∼20% (5), when compared with scores from uninfected patients. This was demonstrated in a cohort of 937 individuals, predominantly second referral Italian patients; however, it was not observed in second referral German patients (5). Notably, the difference observed in the second referral patients has been recently demonstrated by a prospective study performed on a cohort of ∼400 Chinese patients, further validating the modified UPOINTS system (16). In addition, the mean total NIH-CPSI symptom scores in the Chinese cohort were increased by 20–25% in patients with a positive infection domain of the UPOINTS system, including a sexual dysfunction domain (16).

Since this evidence suggests that the presence of pathogens is correlated with the deterioration of CP/CPPS symptoms, the present subset study analyzed in greater detail the symptom profile of a cohort of patients with CP/CPPS who had previously been included in two studies aimed at investigating the NIH-CPSI in patients with CP/CPPS, and at validating the UPOINT algorithm (5,6).

The patients were divided into two groups, depending on whether the prostate-specific specimens of patients symptomatic for CP/CPPS were positive or negative for cultures. The median NIH-CPSI scores, as well as pain and QoL subdomain scores, were significantly higher in the patients with a positive UPOINT infection domain, compared with patients with no evidence of infection (Table I). An exception was represented by the voiding score of NIH-CPSI, which is known to be the least responsive among all NIH-CPSI domains.

Despite the large size of the present patient population, which made the clinical relevance of the statistical significance of these differences debatable, the statistically significant, 4-point difference observed between the ‘infection’ and the ‘non-infection’ groups (median scores, 24 versus 20 points; Table I) corresponded to the commonly accepted minimum value of a clinically appreciable difference in total NIH-CPSI scores (17).

Notably, the analysis of symptom score distributions resulted in a right-shift of the curve representing pain symptoms and total NIH-CPSI scores in patients with evidence of prostatic infection (Fig. 2A and D). This evidence suggested the existence of populations showing different degrees of symptom severity, depending on the presence/absence of a concomitant prostatic infection.

Data from of a historical cohort of 157 Hellenic patients from our research group supported this finding: Patients showing the presence of pathogens in prostate-specific specimens (assessed with the Meares and Stamey segmented test) had higher NIH-CPSI symptom scores, compared with patients without evidence of infection (NIH-CPSI total score median difference: 7 points; P<0.0001, Mann-Whitney-Wilcoxon test; analysis not shown).

Subsequently, it was investigated whether it was possible to reproduce differential symptom scores in the two populations (infected versus non-infected) when the patient population was divided according to the six phenotypic clusters of patients with CP/CPPS, as described by Davis *et al* (10). The ‘prostate infection-related sexual dysfunction’ cluster, including the highest proportion of patients with evidence of infection (80%), scored the highest number of points of the NIH-CPSI among all the clusters. To assess the influence of the infection domain on the severity of the symptoms exhibited by the patients, all subjects with evidence of infection were withdrawn from the prostate ‘infection-related sexual dysfunction’ cluster. This modified cluster showed symptom scores significantly less severe than those of the original cluster. Furthermore, the scores were comparable with those of the five other clusters.

Another recent study investigated the separation of patients with CP/CPPS into distinct phenotype clusters (18). According to this study, it was possible to divide the patients into two major clusters. One cluster included patients with increased frequencies of U, O and T domains, while the other showed a high frequency of P, I and N domains of UPOINT [notably, the ‘UOT’ cluster in our study cohort represented the most prominent group of patients (data not shown)]. Separation of the present patient population into the ‘UOT’ and ‘PIN’ clusters resulted in significantly higher NIH-CPSI symptom scores in the latter cluster (median total NIH-CPSI scores: 29 versus 25 in the ‘UOT’ cluster; P<0.05, Mann-Whitney Wilcoxon test), which contained higher proportions of subjects showing evidence of infection (detailed analysis not shown). Furthermore, when patients with evidence of infection were withdrawn from the cluster, NIH-CPSI scores decreased significantly [median total NIH-CPSI score: 24 (detailed analysis not shown)].

In combination, the results of the present study strengthened the hypothesis that the presence of pathogens in the prostate gland may exert a significant effect on the clinical presentation of patients affected by CP/CPPS, and that the UPOINTS infection domain represents an important determinant of the severity of CP/CPPS symptoms in clusters of patients characterized by a positive infection domain of the UPOINTS system. This evidence implies that, if pathogens are partly responsible for a deterioration of symptoms in CP/CPPS, antibacterial agents may represent an important therapeutic tool for the treatment of patients showing evidence of prostate colonization by uropathogens or by other suspiciously pathogenic bacteria. A retrospective analysis is in progress and a prospective study is being planned to assess the efficacy of antibacterial therapy in this specific category of patients.

## Figures and Tables

**Table I.** Median values of the NIH-CPSI total score and symptom domain subscores, and statistical analysis of differences between cohorts of patients with category III CP/CPPS with or without evidence of prostatic infection, defined according to NIH criteria (7).

NIH-CPSI score	Total patient population		Patients with evidence of infection		Patients without evidence of infection	
	Median	Upper and lower quartiles	Median	Upper and lower quartiles	Median	Upper and lower quartiles
1 - pain domain	9	7–12	11	8–14	9^a^	7–11
2 - voiding symptom domain	4	2–6	4	2–7	4^b^	2–6
3 - QoL impact domain	8	5–9	8	6–10	7^a^	5–9
Total score (1+2+3)	21	16–26	24	18–29	20^c^	15–24

a P<0.01 vs. patients with evidence of infection, Mann-Whitney-Wilcoxon test;

b P>0.05 vs. patients with evidence of infection, Mann-Whitney-Wilcoxon test;

c P<0.001 vs. patients with evidence of infection, Mann-Whitney-Wilcoxon test. NIH-CPSI, National Institutes of Health-Chronic Prostatitis Symptom Index; CP/CPPS, chronic prostatitis/chronic pelvic pain syndrome; QoL, quality of life.

**Table II.** Median values (and interquartile range) of the NIH-CPSI total score and symptom domain subscores, and statistical analysis of differences between cohorts of patients with category III CP/CPPS divided by cluster according to Davis *et al* (10). Median scores of group ‘1^*^’, a subgroup of cluster 1 devoid of patients showing evidence of infection, defined according to NIH criteria (7), are also presented.

NIH-CPSI score	Cluster classification						
	1	1^*^	2	3	4	5	6
1 - pain domain	12 (5)	9^a^ (4)	9^a^ (5)	9^a^ (5)	10^a^ (4)	10^a^ (4)	9^a^ (5)
2 - voiding symptom domain	6 (3.75)	5^a^ (2.00)	4^a^ (4.00)	4^a^ (4.00)	6^b^ (2.00)	4^a^ (4.00)	5^a^ (4.00)
3 - QoL impact domain	9 (4)	8^a^ (3)	8^a^ (3)	8^a^ (3)	9^b^ (4)	8^a^ (4)	8^a^ (3)
Total score (1+2+3)	28 (9.75)	22^a^ (9.00)	21^a^ (10.00)	22^a^ (9.00)	24^a^ (7.50)	22^a^ (10.00)	22^a^ (9.00)

Cluster classification, according to Davis *et al* (10): 1, prostate infection-related sexual dysfunction; 1^*^, same as ‘1’, but with the withdrawal of patients showing evidence of infection; 2, widespread symptoms; 3, psychosocially complex lower urinary tract symptoms (LUTS) with sexual dysfunction; 4, psychosocially complex LUTS without sexual dysfunction; 5, muscle tenderness/fibromyalgia-like symptoms; 6, uncomplicated LUTS.

a P<0.01 vs. cluster 1, Mann-Whitney-Wilcoxon test;

b P<0.05 vs. cluster 1, Mann-Whitney-Wilcoxon test. NIH-CPSI, National Institutes of Health-Chronic Prostatitis Symptom Index; CP/CPPS, chronic prostatitis/chronic pelvic pain syndrome; QoL, quality of life.
